# Supplementary material for: AIDS Drug Assistance Program disenrollment is associated with loss of viral suppression beyond differences in homelessness, mental health, and substance use disorders: An evaluation in Washington state 2017–2019
Source: PLoS One. 2023 May 4;18(5):e0285326. doi: 10.1371/journal.pone.0285326 (PMC10159195; doi:10.1371/journal.pone.0285326)
Supplement: Attachment — Submitted filename: Response to Reviewers.docx [file pone.0285326.s001.docx]

January 31, 2023

To the Reviewers and Editors of PLOS One,

Thank you for taking the time to review our manuscript and giving us an opportunity to improve our work. We have made major revisions detailed in the below table. As requested we would also like to present our updated funding statement:

*JCD and CMK's work was supported by the University of Washington / Fred Hutch Center for AIDS Research, an NIH-funded program under award number AI027757 which is supported by the following NIH Institutes and Centers: NIAID, NCI, NIMH, NIDA, NICHD, NHLBI, NIA, NIGMS, NIDDK. There was no additional external funding received for this study*

Please do not hesitate to contact me if you have any questions.

Steven Erly

steven.erly@doh.wa.gov

| Reviewer 1 Comment | Revision |
| --- | --- |
| First, please add a “Table 1” of descriptives of the main study sample. Some of this information is in Supplementary Table 1. I recommend moving that to the main text and adding a column to provide descriptives on the full sample. In the Supplementary Table 1, the prevalence ratio is less intuitive for illustrating the differences between the two groups. As your reader, I am unsure how to interpret this column to quickly understand how characteristics differ between groups. Is there an easier way to present p-values showing differences between groups? If you prefer this format, please explain in the footnote and text how to interpret. | We have moved the supplementary table to the main text and have added a column for individuals ruled ineligible so that the entire population is represented (Table 1). We have removed the prevalence ratio, as this measure of association was not pertinent to describing the entire cohort. |
| Second, I recommend a flow chart or other visualization to explain the sample design. Starting on line 136, the authors explain that each client contributed one recertification opportunity. If recertification were required every 6 months during the study period (per federal rules in place during the 2017-2019 study period), a client could potentially contribute multiple observations. It would be useful to have a better understanding of the time period of the study (are most observations from 2017 in the initial event?) and explanation for only considering one observation per study participant. | In lieu of a flow chart, we expanded the text in the results section to illustrate the composition of the sample.  Results, First Paragraph: “From 2017-2019, 5480 clients were enrolled in ADAP, of whom 5237 (96%) met our inclusion criteria; 3006 (55% of ADAP enrollees) remained in ADAP through the end of 2019 and 2231 (40% of ADAP enrollees) received a viral load or lived in WA for 12 months after leaving ADAP.”  We have added an explanation of why we used only one observation per study participant.  Methods, primary analysis, paragraph 2: “Each ADAP client contributed one recertification opportunity to the model; although individual clients may have encountered multiple recertification opportunities during our study period, we chose to analyze only a single event to ensure that we did not re-use individual viral load test results across observations.” |
| Third, a challenge with the HIV VL data is that in practice, VL testing is not always done precisely every 6 months. The VL tests can be missing for multiple reasons: falling out of care (which the authors code as not virally suppressed), having the tests done out of state and not reported to Washington’s surveillance system (for example, for persons who spend winter months in a different state), or because the patient is stable on their medications thereby receiving less frequent VL testing than the clinical guidelines (a comment I have heard anecdotally from clinicians). The authors already have a sensitivity analysis that excludes participants without a VL measurement before or after recertification. It would be useful to have more details on the timing of VL measurements in the data such as the percent of participants with 1+ or 2+ VL tests per year, the average and range in the time in between disenrollment & recertification events and VL testing, and comparison of sub-populations with and without regular VL testing. Please also discuss in the limitations how the timing of VL tests in relation to the time of recertification & disenrollment events might bias findings. | We have added a table to the result section containing this information (Table 3) and have included the following text.  Results, first section, final paragraph: “For those who received viral load testing within a year recertification, the median time to testing was 3 months and was the same for those who recertified, dropped, or were ruled ineligible. Full information about frequency of viral load testing can be found in Table 3.”  We have also expanded our limitations section discussing missing viral loads to incorporate this point.  Discussion, paragraph 4: “Our study had several limitations. The first is related to our measurement of viral suppression. Some people who have left Washington state or who only require infrequent viral load monitoring as may be misclassified as virally unsuppressed. However, our sensitivity analysis that includes only people who have labs reported in Washington confirms the presence of a risk difference even in a situation where this misclassification is not possible” |
| Reviewer 2 Comment |  |
| 2. Methods, lines 84-87: The inclusion criteria as described for this study is a little confusing. How is it known whether a client lives in Washington State for 12 months after leaving ADAP if they drop out of care? Is there active follow-up of those who drop out? Since this analysis uses surveillance data, it would seem that the residency status (Washington residence versus another state) of someone who disenrolls and does not have any contact with the providers in Washington State for over a year would be difficult to ascertain. | We have updated this section to clarify how address is ascertained.  Methods, paragraph 1: “Residency in Washington state was inferred from subsequent laboratory reports or interstate deduplication efforts based on Soundex that occur when a person has not received a lab in Washington for 18 months.” |
| 3. In addition to the important message about disenrollment and viral suppression, there may be a message about ineligibility and viral suppression worth mentioning in the Discussion, since those who became ineligible for ADAP also had substantial decreases in suppression (albeit in a smaller subset). However, some additional context may be necessary to better understand this (see my minor comment #1 below). | We have added a section to the discussion about ineligibility. Although we do not have specific information about reasons for ineligibility, the number of possibilities are small and we discuss these in the section.  Discussion, Paragraph 2: “It is noteworthy that there was also significant decrease in viral suppression among those who were ruled ineligible from ADAP. This population is presumed to have alternative mechanisms to pay for healthcare (an income>425% FPL or Medicaid), but these results suggest that disruption of or change to medical coverage may still adversely impact this population.” |
| 4. The comment in the Discussion that Washington is a Medicaid expansion state is an important one given that the inferences identified here could be exacerbated in a non-Medicaid expansion state. In fact, based on Table 1, “other public insurance” is the second largest group after “private insurance”. Can the authors clarify what type of insurance this is? Is it mostly Medicaid beneficiaries? | We have added this information to the methods section to clarify  Methods, Study Design and Data Sources, Final Paragraph:  “For insurance type, “other public insurance” primarily consists of Medicare plans, but also includes VA and Indian Health Service coverage. Individuals with Medicaid are generally not eligible for ADAP (apart from those with dual Medicare-Medicaid).” |
| 1. Is there information on what the reasons for disenrollment and/or ineligibility among ADAP enrollees in Washington State, and how they are distributed? E.g., how often do ADAP clients lose eligibility because they obtain health insurance through a new job? Some context may be helpful to include in the Discussion if this information is available (although it may not be available). | See Major Comment 3. |
| 2. Introduction, first sentence: The authors state that ADAPs are “a critical part of federal plans to reduce HIV incidence by 90% by 2030”. One assumes that this is because of the concept of “treatment as prevention” – this could be made clearer. | Introduction, first sentence: modified sentence to “and a critical part of the Treat Pillar in federal plans to reduce HIV incidence by 90% by 2030” |
| 3. Introduction, line 60: The authors note that “individual programs now have the authority to modify the recertification requirement”. It would be helpful to insert the word “state” before the word “programs” to clarify and emphasize that ADAPs are administered by states. | Introduction, paragraph 2: changed word “individual” to “state” |
| 4. Methods, line 107: if multiple VLs are recorded for a client before and after recertification opportunities, which ones are used in the analysis? | Methods, Study Design and Data Sources, Paragraph 4: added “is” |
| 5. Methods, line 108: the word “is” is missing after “Viral suppression” | Methods, Study Design and Data Sources, Paragraph 4: added “of” |
| 6. Methods, line 109: the word “of” is missing after “reduced risk” | Methods, Study Design and Data Sources, Paragraph 4: added “of” |
| 7. Methods, line 156: consider replacing the word “estimate” with “estimand” | Methods, Qualitative Bias Analysis, Paragraph 1: Changed “Estimate” to “Estimand” |
| 8. Methods, lines 171-172. Please clarify whether national or Washington State MMP data were used to estimate the bias parameters. | Methods, Qualitative Bias Analysis, Paragraph 1: Specified “Washington” MMP Data. |
| Journal Requirements |  |
| 1. Please ensure that your manuscript meets PLOS ONE's style requirements, including those for file naming. The PLOS ONE style templates can be found at | We have updated the files to match the linked style guides |
| 2. In your ethics statement in the manuscript and in the online submission form, please provide additional information about the patient records used in your retrospective study. Specifically, please ensure that you have discussed whether all data were fully anonymized before you accessed them. | Added statement: “Data was anonymized during analysis, but not before access as the study team is responsible for data collection.” |
| 3. Thank you for stating in your Funding Statement:  “JCD and CMK's work was supported by the University of Washington / Fred Hutch Center for AIDS Research, an NIH-funded program under award number AI027757 which is supported by the following NIH Institutes and Centers: NIAID, NCI, NIMH, NIDA, NICHD, NHLBI, NIA, NIGMS, NIDDK. https://depts.washington.edu/cfar/?q=home”  Please provide an amended statement that declares *all* the funding or sources of support (whether external or internal to your organization) received during this study, as detailed online in our guide for authors at http://journals.plos.org/plosone/s/submit-now.  Please also include the statement “There was no additional external funding received for this study.” in your updated Funding Statement.  Please include your amended Funding Statement within your cover letter. We will change the online submission form on your behalf. | Added updated funding statement to cover letter |
| 4. Please include captions for your Supporting Information files at the end of your manuscript, and update any in-text citations to match accordingly. Please see our Supporting Information guidelines for more information: http://journals.plos.org/plosone/s/supporting-information. | We have moved the supporting information to the main text at the request of the reviewers |
