# Supplementary material for: AIDS Drug Assistance Program disenrollment is associated with loss of viral suppression beyond differences in homelessness, mental health, and substance use disorders: An evaluation in Washington state 2017–2019
Source: PLoS One. 2023 May 4;18(5):e0285326. doi: 10.1371/journal.pone.0285326 (PMC10159195; doi:10.1371/journal.pone.0285326)
Supplement: Attachment — Submitted filename: Response to Reviewers.docx [file pone.0285326.s002.docx]

April 10th, 2023

To the Reviewers and Editors of PLOS One,

Thank you for taking the time to review our manuscript and giving us an opportunity to improve our work. We have made revisions detailed in the below table. Please do not hesitate to contact me if you have any questions.

Steven Erly

steven.erly@doh.wa.gov

| Reviewer 2 Comment |  |
| --- | --- |
| 1. Results section: The overall eligible N of 5237 does not match the sum of the component categories (1336 disenrolled, 896 ineligible, 3006 continuously enrolled). It is off by 1. Should the N be 5238? | Thank you for catching this; this was indeed a typo. The N is now 5238, which is consistent with the other numbers in the document. |
| 2. Table 1: The “race” category should be changed to “race/ethnicity” since Hispanic is an ethnicity, not a race. | Agreed. We have made this change to Table 1, the footnotes of Table 2, and line 149 of Methods. |
